# Branch Retinal Artery Occlusion Secondary to Infective Endocarditis

**DOI:** 10.1155/2021/8828876

**Published:** 2021-01-15

**Authors:** Yixu Zheng, Ke Xiong

**Affiliations:** Department of Ophthalmology, Nanfang Hospital, Southern Medical University, Guangzhou, Guangdong, China

## 1. Introduction

Systemic disease is usually responsible for retinal ischaemic events in patients under 30 years of age [1, 2]. We report the case of a young woman with branch retinal artery occlusion secondary to infective endocarditis.

## 2. Case Report

A healthy 24-year-old female presented with sudden painless decrease in vision in the lower right quadrant of her left eye after awakening from her nap that afternoon. She was well, apart from an episode of dizziness after bathing during the previous 2 weeks. The episode had started 1 week after the patient had got acute tonsillitis. She denied associated symptoms of headache, fever, recreational drug abuse, and any other medical history.

**Figure 1:**
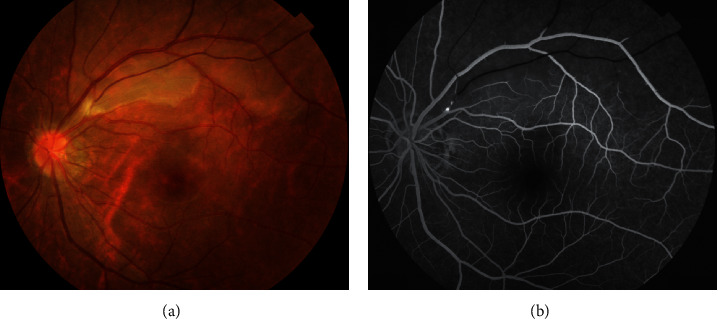
Fundus photograph of the left eye demonstrated plaque and mild edema of the supertemporal retina. Corresponding fluorescein angiogram highlighted hyperfluorescence and nonfilling of the supertemporal artery.

**Figure 2:**
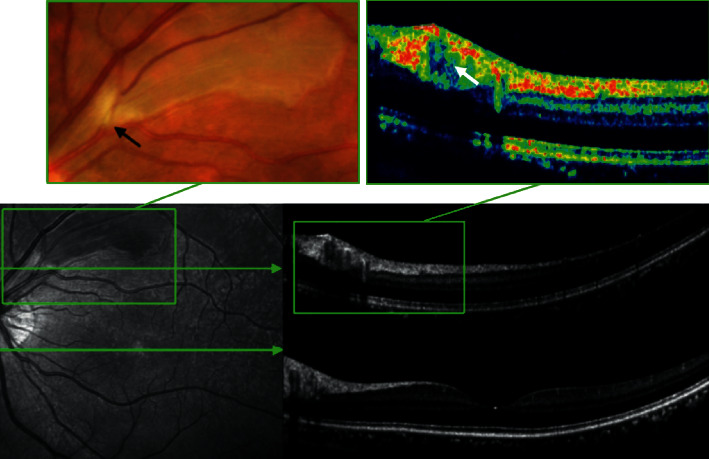
Optical coherence tomography revealed increased thickness of the inner retinal layers and normal fovea. Blown-up fundus photograph revealed a plaque within the supertemporal artery (black arrow). Blown-up optical coherence tomography (pseudo-color) revealed blocked artery with highly reflective material (white arrow) in the corresponding site.

Examination of the affected eye revealed a vision of 20/20. Anterior segment examination was unremarkable in both eyes. Dilated fundus exam of the left eye demonstrated a region of retinal edema along the supertemporal arcade extending just above the macula. Confrontation visual field revealed a loss of inferior visual field in the left eye. The fundus photograph and fluorescein angiogram from the day after presentation are shown in Figure 1. An optical coherence tomography showed supertemporal inner retinal edema and visualized the intra-arterial embolus, as shown in Figure 2.

**Figure 3:**
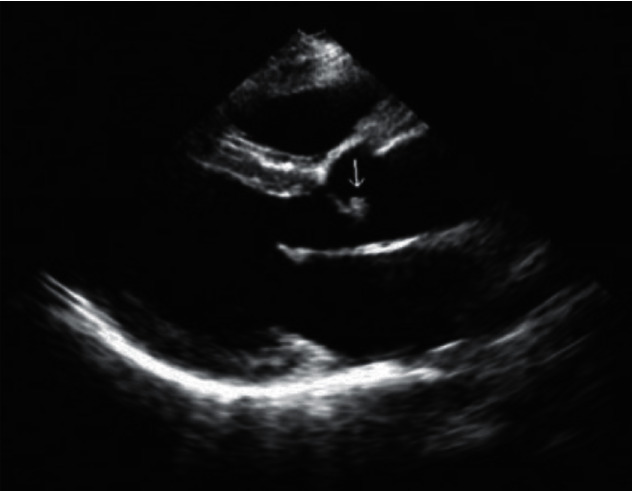
Transthoracic echocardiogram demonstrated the vegetation locating in the aortic valve (white arrow).

**Figure 4:**
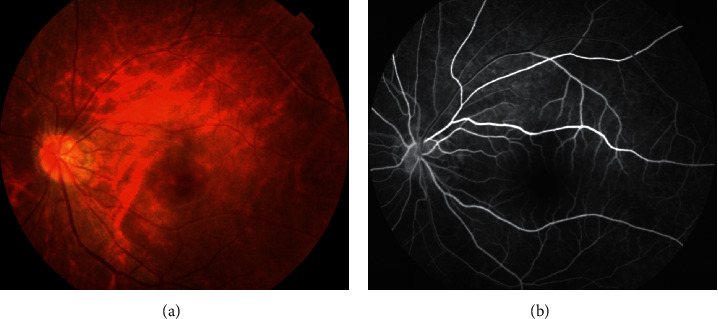
Fundus photograph and fluorescein angiogram at 4 weeks demonstrated returning to normal.

Her retinal appearance and symptoms prompted referral to the cardiology team, which admitted her that day. Urgent erythrocyte sedimentation rate, C-reactive protein, and complete blood count were all normal. However, the transthoracic echocardiography revealed moderate-to-severe aortic valve regurgitation, with thickening and sign of vegetation, as shown in Figure 3 [3]. Blood cultures yielded *Streptococcus viridans*. The patient was diagnosed with infective endocarditis, and intravenous antibiotics were started immediately. Her visual symptoms subsided after medical treatment. The supertemporal retinal edema had completely resolved. Confrontation fields showed improvement in the prime inferior visual field defect of the left eye. Optical coherence tomography line scan showed no hyper-reflectivity. Her left fundal appearance and fluorescein angiogram after 4 weeks is shown in Figure 4.

## 3. Discussion

Branch retinal artery occlusion (BRAO) is a rare diagnosis in the population under the age of 30 years [1, 2]. BRAO presents as an acute painless loss of visual field in the distribution of the occluded artery. Multiple mechanisms exist that cause arterial occlusion in the retina. Ordinarily, BRAO occurs secondary to an embolus. Emboli typically originate within vessels upstream where they dislodge and travel within the circulatory system to ultimately become lodged downstream in a vessel with a smaller lumen [4–6]. The most common are cholesterol emboli from aortocarotid atheromatous plaques, platelet fibrin emboli from thrombotic disease, and calcific emboli from cardiac valve disease [7].

The fundus shows a whitish, retinal edema in the distribution of the occluded artery, and the corresponding fluorescein angiogram highlights nonfilling of the occluded artery. Optical coherence tomography (OCT) is increasingly being used in various retinal diseases due to its user friendliness and high-resolution imaging capabilities [8–10]. In this case, a vertical scan reveals hyper-reflectivity and thickness of inner retinal layers in the ischaemic retina. OCT could also show the plaque as an area of high reflectivity within the lumen of the blocked artery. It is a patient with acute painless quadrantic loss of visual field due BRAO. Given the patient's history of dizziness and tonsillitis, cardiac valve disease is most likely. Using transthoracic echocardiogram and *Streptococcus viridans*-positive blood cultures, the patient was diagnosed with infective endocarditis without fever and with loss of visual field as the only symptom [5].

BRAO due to infective endocarditis is extremely rare. The presence of BRAO in the young adult population should prompt the consideration of possible infective endocarditis [1]. In spite of infective endocarditis having many different forms of presentation, a high clinical suspicion is often required to reach the diagnosis. The ophthalmological pathology can help to uncover serious underlying medical conditions, which may reduce mortality and morbidity in these patients [5, 6].
